# Construction of *Lactobacillus casei* ghosts by Holin-mediated inactivation and the potential as a safe and effective vehicle for the delivery of DNA vaccines

**DOI:** 10.1186/s12866-018-1216-6

**Published:** 2018-07-28

**Authors:** Rui Hou, Muzi Li, Tingting Tang, Ruichong Wang, Yijing Li, Yigang Xu, Lijie Tang, Li Wang, Min Liu, Yanping Jiang, Wen Cui, Xinyuan Qiao

**Affiliations:** 1 Heilongjiang Key Laboratory for Animal Disease Control and Pharmaceutical Development,Department of Preventive Veterinary, College of Veterinary, Northeast Agricultural University, 59 Mucai Street, Harbin, 150030 China; 2 Institute for Radiological Protection, Heilongjiang Province Center for Disease Control and Prevention, 40 Youfang Street, Harbin, 150030 China

**Keywords:** Bacterial ghost, *Lactobacillus casei*, DNA vaccine, Phage

## Abstract

**Background:**

Bacterial ghosts (BGs) are empty bacterial cell envelopes generated by releasing the cellular contents. In this study, a phage infecting *Lactobacillus casei ATCC* 393 (*L. casei* 393) was isolated and designated Lcb. We aimed at using *L. casei* 393 as an antigen delivery system to express phage-derived holin for development of BGs.

**Results:**

A gene fragment encoding holin of Lcb (hocb) was amplified by polymerase chain reaction (PCR). We used *L. casei* 393 as an antigen delivery system to construct the recombinant strain pPG-2-hocb/*L. casei* 393. Then the recombinants were induced to express hocb. The immunoreactive band corresponding to hocb was observed by western-blotting, demonstrating the efficiency and specificity of hocb expression in recombinants. The measurements of optical density at 600 nm (OD_600)_ after induction showed that expression of hocb can be used to convert *L. casei* cells into BGs. TEM showed that the cytomembrane and cell walls of hocb expressing cells were partially disrupted, accompanied by the loss of cellular contents, whereas control cells did not show any morphological changes. SEM showed that lysis pores were distributed in the middle or at the poles of the cells. To examine where the plasmid DNA was associated, we analyzed the *L. casei* ghosts loading SYBR Green I labeled pCI-EGFP by confocal microscopy. The result demonstrated that the DNA interacted with the inside rather than with the outside surface of the BGs. To further analyze where the DNA were loaded, we stained BGs with MitoTracker Green FM and the loaded plasmids were detected using EGFP-specific Cy-3-labeled probes. Z-scan sections through the BGs revealed that pCI-EGFP (red) was located within the BGs (green), but not on the outside. Flow cytometry and qPCR showed that the DNA was loaded onto BGs effectively and stably.

**Conclusions:**

Our study constructed *L. casei* BGs by a novel method, which may be a promising technology for promoting the further application of DNA vaccine, providing experimental data to aid the development of other Gram-positive BGs.

## Background

Bacterial ghosts (BGs) are nonliving cell envelopes of bacteria which are generated by lysing the cell walls and releasing the cytoplasmic contents through channels in the cell envelope. BGs are generally produced by expressing the cloned lysis gene E of bacteriophage ΦX174 in Gram-negative bacteria [1]. When the E protein is induced and expressed under suitable conditions, specific transmembrane channels of 40-200 nm form in the bacterial cell walls. Under the influence of osmotic pressure, the cytoplasmic contents are released [2]. E-mediated lysis has been applied in many kinds of Gram-negative bacteria including *Escherichia coli* [3–5], *Helicobacter pylori* [6, 7], *Salmonella enteritidis* [8–11], *Salmonella gallinarum* [12, 13], *Salmonella typhi* [14], *Edwardsiella tarda* [15, 16], *Aeromonas hydrophila* [17], *Vibrio anguillarum* [18], *Chlamydia trachomatis* [19], *Yersinia enterocolitica* [20], *Flavobacterium columnare* [21], *Haemophilus parasuis* [22] and so on.

Since BGs still possess complete antigen structures on the bacterial cell surface [23–25], they can be used directly as vaccines. BGs are also good vehicles for loading biomacromolecules such as antigens, drugs, and DNA [26–29]. Furthermore, BGs are also endowed with intrinsic adjuvant properties as they contain immunostimulating compounds such as peptidoglycan [30]. Another advantage is that the space inside a BG is large, so that multiple epitopes can be presented simultaneously [31–34]. However, there are still some challenges in preparing BGs. BGs reported till now are all prepared with pathogenic bacteria. The current technology used to prepare BGs cannot lyse 100% of the bacteria. If pathogenic bacteria are used to prepare BGs, there is a risk of infection. Therefore, it is essential to choose a safe bacterial host to develop BG.

Lactic acid bacteria (LAB) have been recognized as safe (GRAS) by the American Food and Drug Administration (FDA). For more than 20 years, LAB have been used as potential bacterial carriers to express heterologous proteins in many different fields [35–38]. Specifically, in immunological research, LAB enables immunization via mucosal routes, which is not only more effective for pathogens, which infect hosts through mucosal routes but is also a simpler method than injection. Many researchers have shown that delivery of antigens via LAB may induce not only mucosal but also systemic immune response [39]. The advantage of LAB in immunoprophylaxis and therapy also depends on their resistance to the low pH of gastric juice, which aids in transit through the stomach to reach the immune sites and induce effective immune responses. Moreover, LAB can adhere to the surface of intestinal epithelium, making the immunostimulation more effective and persistent. Furthermore, the components of LAB have adjuvant properties, which can enhance the immune responses induced by the carried antigen. LAB ghosts can be produced by fermentation in large quantities to save time and labor.

In this study, we developed *L.* *casei* ghosts by expressing holin of the *L.* *casei* phage. The method is different from the previous methods of producing BGs. Furthermore, LAB are safe and have no infectivity even though they cannot be lysed completely when they are being used to prepare BGs.

## Methods

### Bacterial strains and plasmids

*Lactobacillus* secretory expression vector pPG-2 which contains the secretion signal peptide gene sequence (ssUSP) and *Lactobacillus casei ATCC* 393 were kindly provided by the Netherlands NIZO Institute. *L. casei ATCC* 393 was cultured anaerobically and statically in MRS (De Man Rogosa Sharpe) medium (Sigma, St, Louis, MO) at 37 °C. The recombinants were cultured in MRS culture medium containing 1% xylose.

### Isolation of *L. casei* phage

In our previous work, a virulent phage against *L. casei ATCC* 393 was isolated from fermented vegetables and designated as Lcb [40]. The phage Lcb was purified and stored in our laboratory.

### Extraction of phage DNA

*L. casei ATCC* 393 was cultured for 12 h and then was added to 100 mL of MRS-Ca-Mg medium. Phage Lcb was inoculated at a MOI of 0.1 when the optical density at 600 nm (OD_600_) of the culture reached about 0.2. The lysate was centrifuged at 8000×g for 10 min. The supernatant was filtered. Then, the filter liquor was treated with DNaseI and RNase A at a concentration of 1 μg/mL at 37 °C for 1 h. The phages were then concentrated with 1 mol/L NaCl and 10% (*w*/*v*) polyethylene glycol 8000 on ice for 1 h, centrifuged at 8000×g for 20 min. The deposit was then resuspended in SM (sodium-magnesium-buffered saline) buffer. Phage DNA was extracted by phenol–chloroform–isoamyl alcohol and then precipitated by isopropanol [40].

### Amplification of holin and construction of the expression plasmid

The pPG-2 plasmid was supplied by Jos Seegers (NIZO, The Netherlands), which is an expression vector with an ssUsp signal peptide. Hocb (encoding holin of Lcb) was obtained and amplified from phage Lcb genome by PCR using forward primer 5’-ACCGCTTGAGACGTGAGAATG-3’ and reverse primer 5’ -GCGACTACCAAAGTGATGAGTTTAG -3’. The gene fragment was 481 bp in length. The PCR amplification was performed as follows: 95 °C for 5 min, 30 cycles at 94 °C for 30 s, annealing at 53 °C for 1 min, and extension at 72 °C for 30 s, followed by an extension at 72 °C for 10 min. The PCR product of the Hocb gene was cutted with *Hin*dIII and *Bam*HI, then connected to the corresponding site of the pPG2 plasmid digested with *Bam*HI and *Hin*dIII to construct the recombinant plasmid (pPG2-Hocb).

Electrotransformation was performed as described previously [38]. Briefly, the ligated plasmid (10 μL) was added into 200 μL of *L. casei* competent cells, blended gently at 4 °C for 3 min, followed by electroporation (25 μF of 2.5 kV/cm). The mixture was added to MRS broth and then incubated at 37 °C for 2.5 h. The recombinants were screened on MRS-agar medium with 10 μg/mL of chloramphenicol (Cm). The recombinants were identified by sequencing.

### Western blot

The recombinants were added to MRS medium with Cm (10 μg/mL) and cultivated at 37 °C for 12 h. Subsequently, the culture was inoculated into MRS medium containing 1% xylose at ratio of 1:10 and cultivated at 37 °C for 36 h. Then the culture was centrifuged at 12000×g for 10 min. The precipitation was followed by washing three times with Tris-Cl (50 mM/L) and lysed with lysozyme (10 mg/mL) at 37 °C for 40 min. The lysates were collected by centrifugation at 10000×g for 10 min and then examined by western blotting to analyze the expression of Hocb protein. The proteins were transferred onto a nitrocellulose membrane and then blocked with phosphate-buffered saline (PBS) containing 5% skimmed milk at 37 °C for 3 h. The immunoblots were washed thrice between any two steps. The immunoblots were incubated with 100 μL (1:100 dilution) of mouse anti-Hocb antibodies (prepared in our lab) in PBS. A horseradish peroxidase (HRP)-conjugated goat anti-mouse IgG (Sigma) was used as a secondary antibody. Detection was developed using the Chemiluminescent Substrate reagent (Pierce, Rockford, IL).

### Preparation and characterization of BGs

The recombinants were inoculated into 5 mL MRS broth containing 1% xylose (or glucose as a negative control). The cultures were induced at 37 °C and the OD_600_ was measured after expression at different time periods (0, 8, 16, 24, 32, 40, 48, 56, 64, 72, 80, and 88 h). The morphology of the *L. casei* BGs was examined by transmission electron microscope (TEM) and scanning electron microscope (SEM) as previously described [12], modified as follows: after lysis, induced and non-induced cells were centrifuged at 4000×g for 10 min and then washed thrice with sterile PBS. The cells were fixed in 2.5% glutaraldehyde for 2 h at 4 °C and then fixed in 1% aqueous osmium tetroxide. This was followed by serial dehydration in ethanol. Samples were observed by SEM (JSM-5200, JEOL, Japan) after coated with a gold-palladium alloy. To prepare negatively-stained samples for TEM, the *L. casei* BG suspension was dropped on copper grids. Extra liquid was removed by blotting paper. Then, the morphology of the BGs was examined by TEM (Hitachi Science Systems, Japan).

### Preparation of fluorescein isothiocyanate (FITC)-labeled linear dsDNA and BGs

The pCI- EGFP (enhanced green fluorescent protein) plasmid (4.7 kb; Clontech, Palo Alto, CA, USA) was extracted by culture of *Escherichia. Coli (E. coli)* by the method described previously [41]. RNA and proteins and lipopolysaccharides (LPS) were removed by RNase I digestion and ammonium acetate precipitation. After precipitation with 70% ethanol, the DNA was dissolved in a TE (Tris-Ethylene Diamine Tetraacetic Acid) buffer and stored at − 20 °C. The purity of pCI-EGFP was analyzed by the ratio of OD_260_/OD_280_. The plasmids with a ratio of 1.9–1.93 were used to load BGs. Subsequently, pCI-EGFP plasmids were labeled with SYBR Green I. Then, the BGs were resuspended in the DNA solution containing HEPES-buffered saline (HBS) buffer at 25 °C for 10 min and then washed three times with HBS.

To further visualize the localization of the loaded pCI-EGFP plasmids, in situ hybridization was performed. Cy-3-labeled probes were obtained by amplifying EGFP using the oligonucleotides 5’-ATGGTGAGCAAGGGCGA-3’ and 5’- TTACTTGTACAGCTCGTCCATG-3’. The BGs were dyed with MitoTracker Green FM (0.5 μg /mL) at 37 °C for 1 h and then washed thrice with HBS [41].

### Laser scanning confocal microscopy

BGs were labeled with the sulforhodamine B and then loaded with the FITC-labeled linear dsDNA. At the same time some BGs were loaded with the SYBR Green I labeled pCI-EGFP. Both of the BGs were examined respectively at 1000 × magnification by a confocal laser scanning microscope (Axioplan; Zeiss, Vienna, Austria). Sulforhodamine B was excited at 563 nm, FITC at 488 nm, and Cy3 at 543 nm. The sulforhodamine B, FITC fluorescence and Cy3 was detected in the wavelength range of 580–620 nm, 565–590 nm and 650–680 nm respectively. The loaded BGs were examined using the confocal laser scanning microscope by z axis sections with a maximal distance of 0.122 μm. The results of scans were recorded [41].

### Quantitative PCR

The BGs loaded with DNA were resuspended in HBS. The BGs loaded with DNA were detected by flow cytometry. The resulting images were analyzed with BD FACSDiva software (USA).

For further quantification, qPCR (quantitative PCR) was performed using SYBR Green I and primers (5’-ATGGTGAGCAAGGGCGA-3’ and 5’- TTACTTGTACAGCTCGTCCATG-3’) for the holin gene. A standard curve was obtained using ten-fold dilutions of the prepared plasmid and qPCR was performed in duplicate. After loading with the plasmid pCI-EGFP, the BGs were washed five times with HBS. The plasmid DNA in the BGs was extracted and quantified by qPCR.

## Results

### Western blotting

**Fig. 1:**
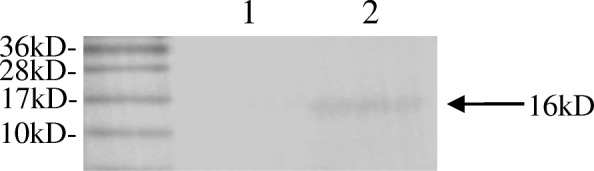
Western-blot analysis of holin expression in recombinant strain. An immunoreactive band was observed for cells grown in the presence of xylose (lane 2), whereas no band was observed in the same cell lysates grown without xylose (lane 1)

For the cells grown in MRS broth containing 1% xylose, the immunoreactive band corresponding to hocb was observed at 16 kD by western blotting (Fig. 1, lane 2). The reactive band was not detected in the control group when the cells were grown in MRS broth without 1% xylose (Fig. 1, lane 1). This indicated that hocb protein expression was induced when the recombinants were cultured in MRS medium containing 1% xylose. The result demonstrated the specificity and efficiency and of hocb protein expression in *L. casei ATCC* 393.

### Preparation and characterization of BGs

**Fig. 2:**
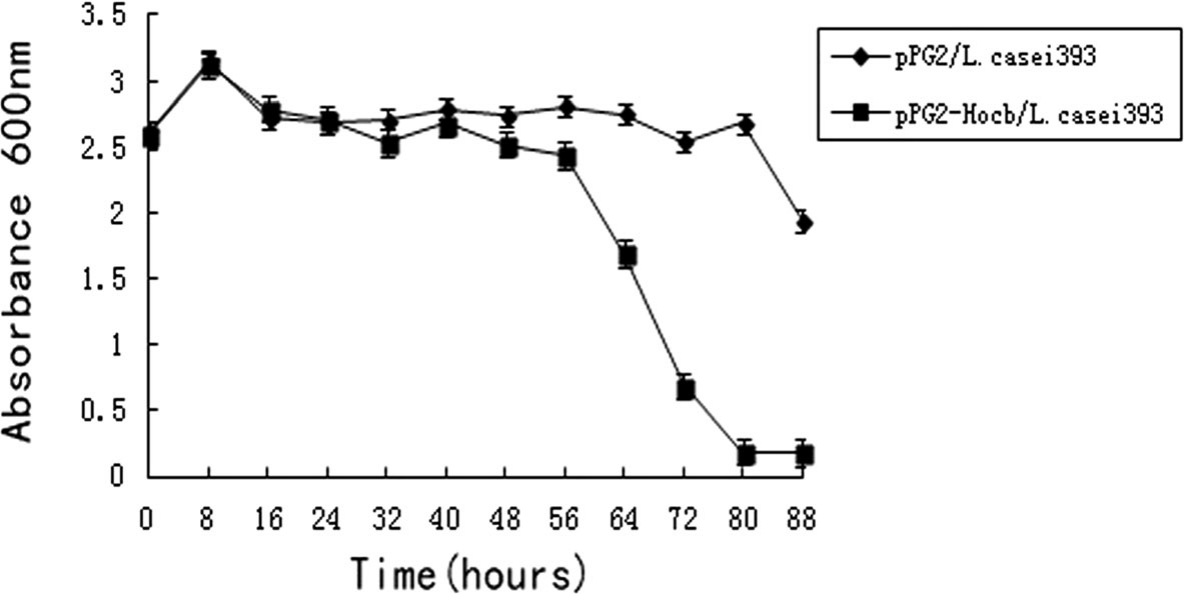
Growth and lysis curves of pPG-2-hocb/L. casei 393 and pPG-2/L. casei 393 following induction. pPG-2-hocb/*L. casei* 393 and pPG-2/*L. casei* 393 were cultured in MRS medium at 37 °C. Expression of the lysis gene hocb was induced with xylose. The optical density (OD_600_) of the culture was monitored at the indicated time points for a period of 88 h after the addition of xylose. OD_600_ values are shown in the graph. *n* = 3; Error bars indicate ± SD

The OD_600_ of pPG-2-hocb/*L. casei* 393 cultures was monitored for a period of 88 h after the addition of xylose (Fig. 2). The OD_600_ of pPG-2-hocb/*L*. *casei* 393 cultures increased during the first 8 h after induction, decreased over next 72 h, and then it remained almost constant until the BGs were collected (Fig. 2). The decline in the OD_600_ curve after the addition of xylose indicated that *L. casei* cells were converted into BGs after the expression of the lysis protein.

**Fig. 3:**
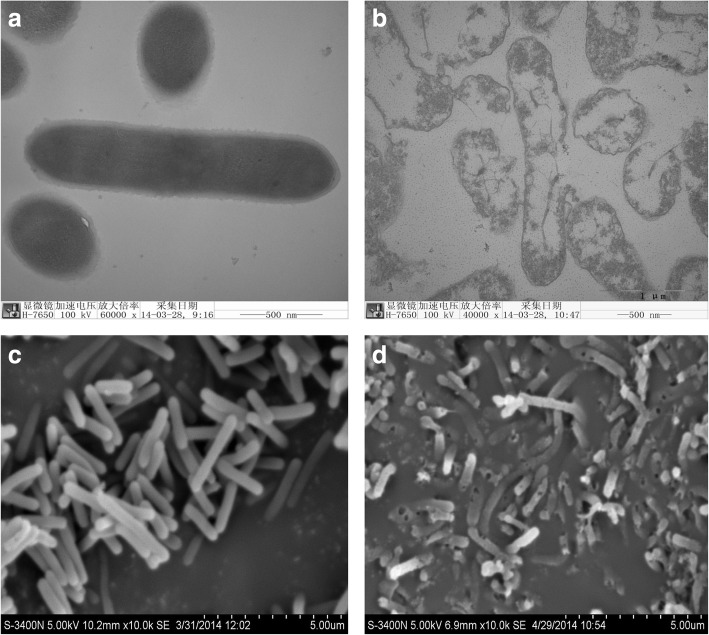
Characterization of *L. Casei* and *L. Casei* ghosts by TEM and SEM. **a** Native *L. Casei* examined by TEM. **b** Loss of cytoplasmic material of *L. Casei* ghosts examined by TEM. Bar = 500 nm. **c** Naive *L. Casei* examined by SEM. **d** *L. Casei* ghosts examined by SEM. Note the presence of transmembrane lysis tunnels

TEM results showed that the cell walls and cytoplasmic membranes of BGs were partially destroyed and the cellular contents were released (Fig. 3b). The control cells did not have any alteration in their morphology and they remained in their distinct forms (Fig. 3a). SEM analysis showed that there were lysis pores present in the envelope of BGs (Fig. 3d) and the lysis pores were distributed mainly in the middle or at the pole of the cells. No changes in morphology were observed for the control cells (Fig. 3c).

### Localization of loaded plasmid and linear dsDNA in BGs

**Fig. 4:**
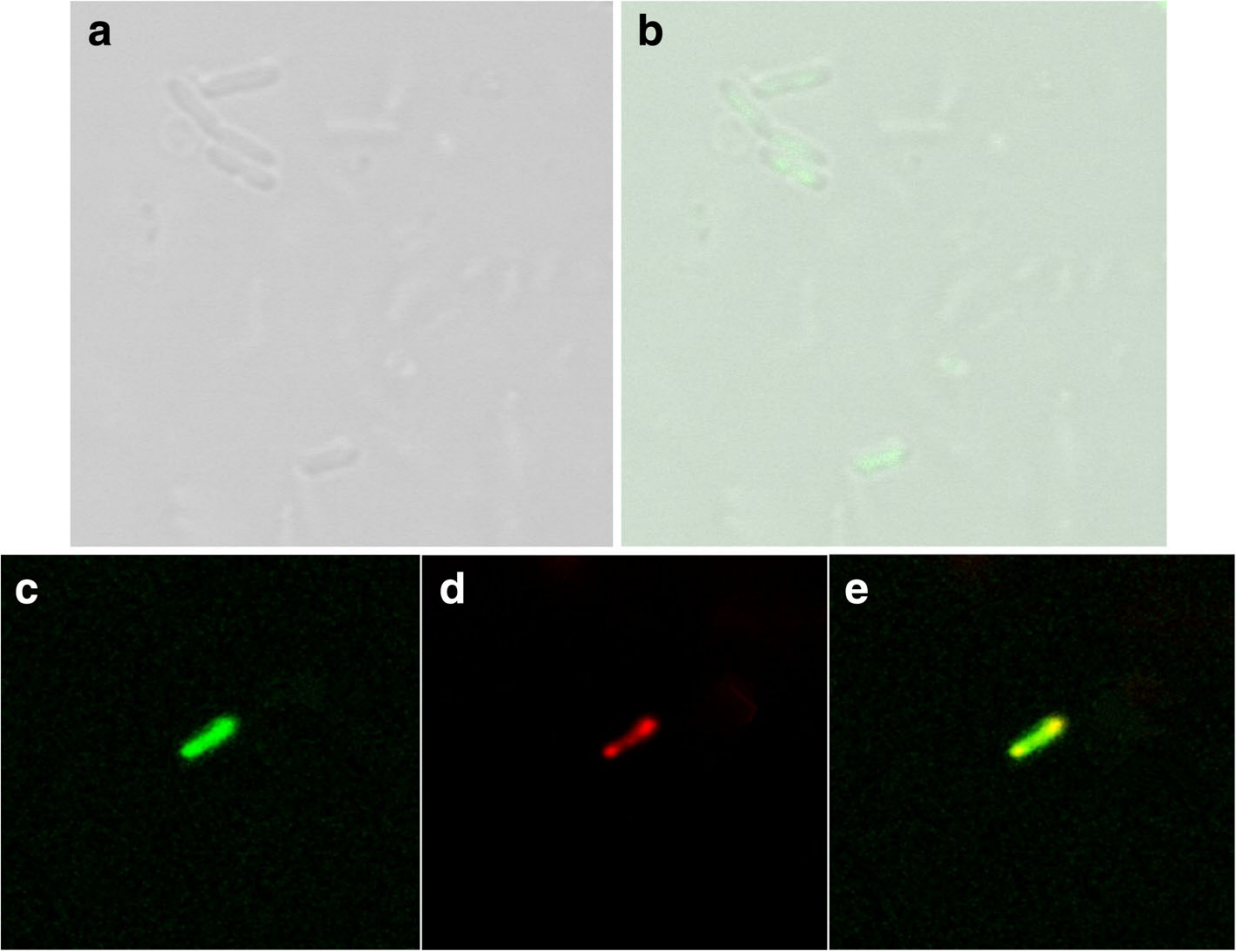
Localization of loaded DNA in *L. Casei* ghosts visualized by confocal laser scanning microscopy. **a** *L. casei 393* ghosts filled with SYBR Green I labeled pCI-EGFP under white light. **b** *L. casei* ghosts filled with SYBR Green I labeled pCI-EGFP. Overlay of differential interference contrast and fluorescent image. The image indicated that the plasmid was filled within the bacterial ghosts. **c** Ghost membranes were stained with MitoTracker Green FM (green). **d** pCI-EGFP was detected by in situ hybridization with Cy3-labeled probes (red) specific for the EGFP. **e** All fluorescence photomicrographs (**c**, **d**) were taken of one middle z-scan section through the middle plane of the loaded bacterial ghosts and subsequently overlaid (**e**). Direct overlays of red and green fluorescent structures are represented by the yellow color

To analyze where the DNA was loaded, we examined the *L. casei* BGs loaded with SYBR Green I labeled pCI-EGFP by confocal microscopy. Green fluorescence was observed in the BGs by confocal microscopy, which confirmed that the SYBR Green I labeled pCI-EGFP was loaded with the inner surface of the BGs. The DNA remained stably associated within the BGs (Fig. 4a and b) after washing three times. This indicated that the DNA interacted with the inside surface of BGs rather than with the outer surface, as DNA entered the BGs only through the lysis tunnels of the cell walls. To further locate the loaded DNA, *L. casei* ghosts were stained with MitoTracker Green FM. The plasmids loaded were then detected by fluorescence in situ hybridization. The result of scans by the confocal laser scanning microscope revealed that pCI-EGFP (red) was located inside of the BGs (green) (Fig. 4c, d, e). These results further confirmed the results obtained with the SYBR Green I labeled pCI-EGFP.

### Quantification of BG-loaded DNA

**Fig. 5:**
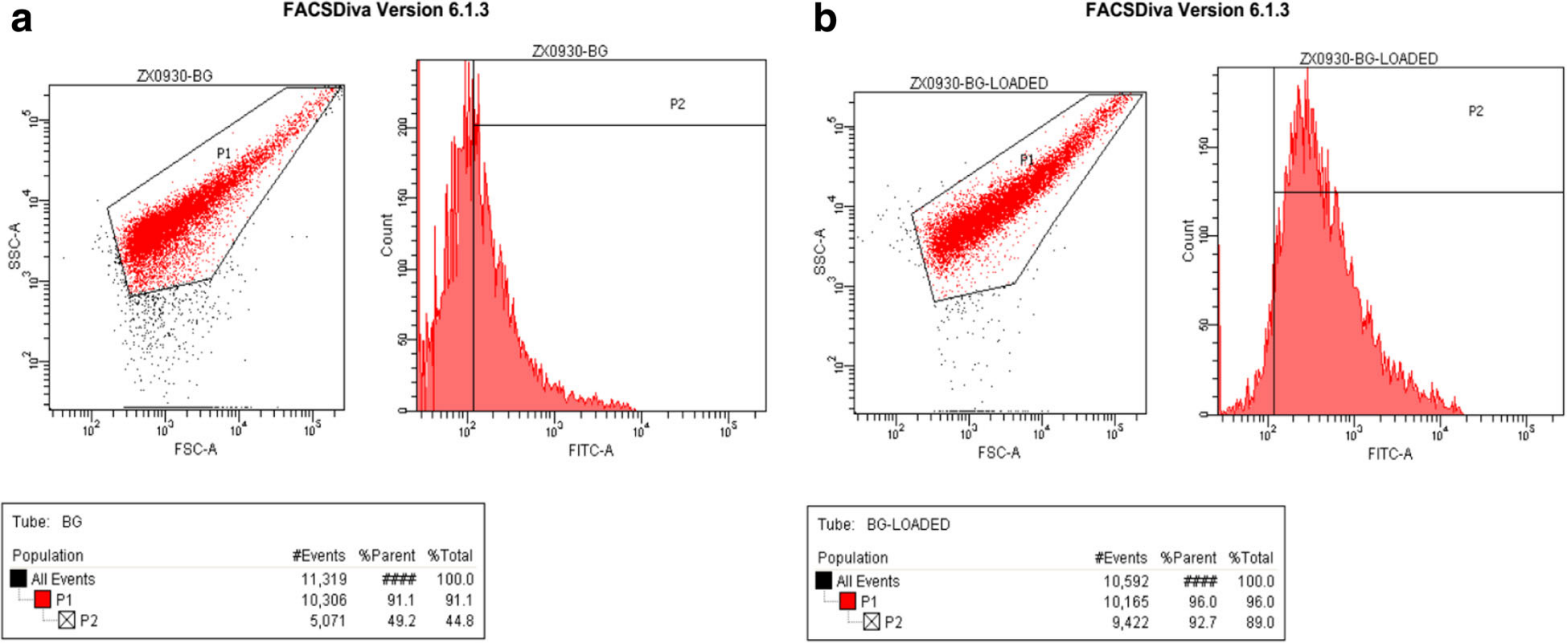
Flow cytometric analysis of the loading efficiency. **a** *L. casei* 393 ghosts unloaded. **b** *L. casei* 393 ghosts loaded with pCI-EGFP plasmid. A distinct shift in the mean fluorescence intensity of the loaded ghosts was observed in comparison with the unloaded ghosts

**Fig. 6:**
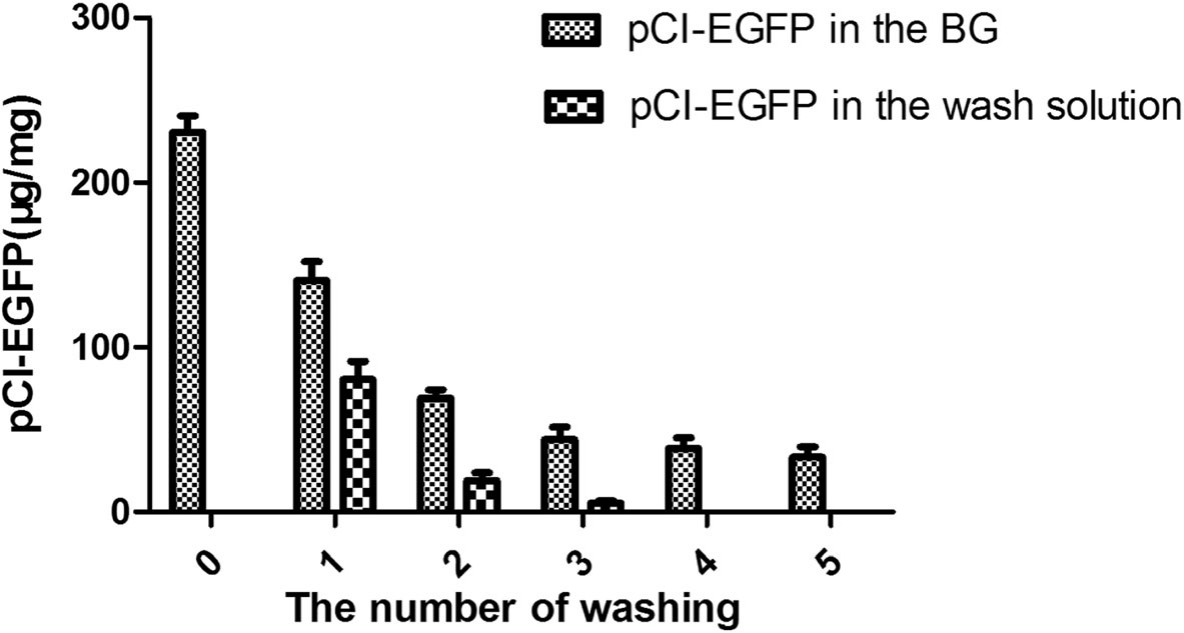
Determination of DNA loaded in the BGs by qPCR. *L. casei* 393 ghosts were loaded with pCI-EGFP plasmid. BGs were washed five times with HBS after loading with pCI-EGFP. DNA in bacterial ghosts and washing solution were extracted respectively and quantified by qPCR. Each point was the mean of quadruplicate measurements ± SD. Each experiment was repeated at least twice

The efficiency of BGs to load DNA was examined by flow cytometry. As shown in Fig. 5, the average fluorescence intensity of loaded BGs shifted distinctly compared with that of unloaded BGs. This indicated that DNA had been loaded in the BGs effectively. Results of qPCR showed that the maximum load is 230.9 μg of pCI-EGFP/mg BGs (Fig. 6). The loading capacity of BGs still reached 44.6 μg/mg after washing three times. Taken together, these results show that DNA can be loaded in BGs effectively and stably.

## Discussion

BGs are empty bacterial cell envelopes created by releasing the cytoplasmic contents through pores in the cell walls. Because BGs retained their original cell shape and surface structures, much of their immunogenicity is retained. If BGs are used as a carrier system to deliver DNA vaccines and drugs to prevent and cure diseases, they will have great application potential. Typically, BGs are prepared by expressing the E gene of phage PhiX174 cloned in bacteria [23]. So far, the dissolution mediated by E protein has been used in many Gram negative bacteria. However, because the cell wall structure of Gram positive bacteria is different from that of Gram negative bacteria, the expression of E gene can not lyse the gram-positive bacteria but kill them. As such, this method cannot be used to develop Gram positive BGs.

In this study, we used *L. casei* to construct BGs by expressing the lysis protein of *L. casei* phage. TEM and SEM analysis showed that we succeeded in preparing *L. casei* BGs using this novel method. TEM analysis showed that there was large space inside of the BGs, which could be loaded with biomacromolecules such as DNA, proteins, and drugs. Up until now, all BGs reported have been created from pathogenic bacteria and research regarding LAB BGs has not been reported. Our study is the first to develop *L. casei* BGs. This method to prepare *L. casei* BGs is different from previous methods reported, which provides experimental data and reference for developing other Gram positive BGs.

The development of DNA vaccines is a major breakthrough in the vaccinology field. DNA vaccines have many advantages compared with traditional vaccines. However, uptake of DNA is difficult because it is easily degraded, and its poor immunogenicity hampers its application [42]. Improving the immune response of DNA vaccines is a key factor in determining immune effect, which is of great significance in theoretical research and production practice. DNA can combine with the inner membrane of BGs non-specifically. Many studies have confirmed that BGs have a strong capacity for loading DNA. BGs not only target the DNA vaccine to antigen presenting cells (APCs), but also act as adjuvants to promote activation and maturation of dendritic cells. HIV gp140 DNA vaccine loaded by *Salmonella typhi* Ty21a BGs were readily taken up by murine macrophage cells so that gp140 could be efficiently expressed. Specific antibody responses in mice immunized with BG-delivered DNA vaccines were significantly higher than those in mice vaccinated with naked DNA [42]. Wen et al. reported that Mannheimia haemolytica BGs were used as carrier to deliver DNA vaccines [14]. In vitro studies demonstrated that one BG could load 2000 copies of plasmid (5 μg DNA/mg BG). Furthermore, vaccination studies demonstrated that more efficient immune responses were stimulated by BG-delivered DNA vaccines than naked DNA. In our study, the results of qPCR showed that the maximum load was 230.9 μg of pCI-EGFP/mg BG. The size of *L. casei* is bigger, therefore the space inside of *L. casei* BG is larger and thus, *L. casei* BGs have stronger capacity to load DNA. Furthermore, *L. casei* BGs are safer than BGs made from pathogenic bacteria even if they cannot be lysed completely. *L. casei* BGs may be used to solve the issues surrounding DNA vaccines, such as poor immunogenicity and high degradability, and improve the level of immune response elicited by them.

## Conclusions

Our study is the first to develop *L. casei* BGs using a novel method, which may be a promising method for developing effective carriers for DNA vaccines. *L. casei* BGs could load DNA effectively and the loaded DNA was connected with the inside of the BGs stably. Our study provides some experimental data and reference for the development of other Gram positive BGs. Furthermore, protein, DNA and drugs can all be loaded in BGs, which offers innovative methods for the design and development of novel vaccines to prevent and treat diseases.

## Abbreviations

APCs: Antigen presenting cells
BGs: Bacterial ghosts
Cm: Chloramphenicol
*E. coli*: E*scherichia. Coli*
EGFP: Enhanced green fluorescent protein
FDA: Food and Drug Administration
FITC: Fluorescein isothiocyanate
GRAS: Generally regarded as safe
HBS: Hepes-buffered saline
HRP: Horseradish peroxidase
HRP: Horseradish peroxidase
*L. casei* 393: *Lactobacillus casei ATCC* 393
lab: Lactic acid bacteria (LAB)
LPS: Lipopolysaccharide
MRS: De Man Rogosa Sharpe
OD_600_: Optical density at 600 nm
PBS: Phosphate-buffered saline
PCR: Polymerase chain reaction
qPCR: Auantitative polymerase chain reaction
SEM: Scanning electron microscope (SEM)
SM: Sodium-magnesium-buffered saline
ssUSP: Secretion signal peptide gene sequence
TE: Tris-Ethylene Diamine Tetraacetic Acid
TEM: Transmission electron microscope
